# The interaction and interference of preformed metal crowns on magnetic resonance imaging: a scoping review with a systematic methodology

**DOI:** 10.1007/s40368-021-00644-z

**Published:** 2021-06-11

**Authors:** O. Sumner, R. Goldsmith, N. Heath, G. D. Taylor

**Affiliations:** 1 School of Dental Sciences, Faculty of Medical Sciences, Newcastle University, Newcastle upon Tyne, UK; 2 Newcastle Upon Tyne Hospital NHS Foundation Trust, Newcastle upon Tyne, UK

**Keywords:** Preformed metal crowns, MRI, Child, Artefacts

## Abstract

**Purpose:**

Preformed metal crowns are widely used to restore primary and permanent teeth. Children may require magnetic resonance imaging (MRI) for diagnosis and monitoring of diseases in the head and neck region. Metallic objects, in the field of view, may compromise the diagnostic value of an MRI. The impact on the diagnostic quality of an MRI in children who have had preformed metal crowns placed has not been assessed. The aim of this systematic review was to evaluate the impact that PFMCs have on MRI imaging quality and thus the overall diagnostic value.

**Methods:**

Electronic searches of the following databases were completed: MEDLINE, EMBASE, Cochrane Library, Web of Science and Open Grey. Primary in vivo studies on children who had at least one preformed metal crown placed and required an MRI investigation were to be included. PRISMA guidelines were followed and screening/data extraction was carried out by two independent calibrated reviewers.

**Results:**

A total of 7665 articles were identified. After removing duplicates, 7062 were identified for title and abstract screening. Thirty-four articles underwent full-text review, of which none met the inclusion criteria. Most common reasons for exclusion were not placing preformed metal crowns (*n* = 16) or in vitro studies (*n* = 12).

**Conclusion:**

No in vivo studies were identified to establish the hypothetical impact preformed metal crowns would have on the diagnostic quality of an MRI in the head and neck region. Decision making needs to be guided on a case by case basis. Further high-quality clinical studies are required.

## Introduction

Preformed metal crowns (PFMC) are widely used in the restoration of primary teeth (Innes et al. 2015), and to a lesser degree permanent teeth (Lygidakis 2010; Taylor et al. 2019). They can be used conventionally, involving caries removal and/or tooth preparation, or using the biological hall technique approach, with good long term success noted (Innes et al. 2015). Their use is popular in the UK (Taylor 2015; Roberts et al. 2018), USA (Crystal et al. 2020) and globally (Hussein et al. 2020), especially in both primary care and specialist paediatric dental care settings.

Magnetic resonance imaging (MRI) is used for diagnosis and monitoring of disease and is especially useful when soft tissues require to be imaged. Specific use within the head and neck region includes seizure disorders, cranial tumours, oro-pharyngeal tumours, and temporomandibular disorders, amongst others (Saunders et al. 2007). In England, within the National Health Service in 2019–2020, 151,710 MRI examinations were performed on children under the age of 14, which is approximately 4% of all MRI examinations performed that year (NHS Diagnostic Imaging Statistics 2020).

An image artefact is a distortion in the image signal intensity caused by a non-anatomical source. Metallic objects are one such source; they disrupt the magnetic fields and cause artefacts including signal loss and distortion (Guermazi et al. 2003). An artefact can make image interpretation difficult, and in extreme cases can render images undiagnostic. This may have an impact on accurate diagnosis or monitoring of disease and subsequently, impact on patient care. Some materials used in dentistry (e.g. stainless-steel orthodontic appliances, cobalt chrome alloys) have been shown to cause artefacts on a MRI image (Hubálková et al. 2006; Tymofiyeva et al. 2013). A recent systematic review investigating the effect of stainless-steel orthodontic appliances on MR images found that in particular, stainless-steel brackets and wires cause distortion and artefact of MR images, rendering some of them undiagnostic (Hasanin et al. 2019). Although, previous in vitro studies suggested that orthodontic bands caused more artefact than orthodontic brackets (Sadowsky et al. 1988). Despite the available evidence showing that orthodontic appliances cause artefact, and subsequently impact the quality of MR images, there is little known about the impact of PFMCs (Sumner and Goldsmith 2019).

Therefore, the aim of this systematic review was to evaluate the impact that PFMCs have on MRI imaging quality, the overall diagnostic value and whether there were any reported safety concerns for patients with PFMCs undergoing an MRI investigation.

## Method

The study protocol was registered with Prospero (CRD42020201753) and reported in line with recommendations of the Preferred Reporting Items for Systematic Reviews and Meta-Analysis (PRISMA) Statement (Moher et al. 2009).

### Search Strategy

Search strategies, shown in Table 1, were developed by one author (GT), with a librarian, and appropriately amended for each database. The included search terms combined MeSH terms and key concepts based on the review question. The review question was ascertained using the PICO framework:

**Table 1:** Search strategies used for each database

*MEDLINE*
1. exp dentistry/or crowns/
2. exp Dental Alloys/
3. preformed metal crown*.mp
4. dentition/or dentition, mixed/ or dentition, permanent/ or molar or tooth, deciduous/or tooth crown/
5. exp Dental Materials/
6. stainless steel crown*.mp
7. 1 or 2 or 3 or 4 or 5 or 6
8. exp Magnetic Resonance Imaging/
9. MRI.mp
10. functional MRI.mp
11. fMRI.mp
12. Tesla.mp
13. 7 T magnet.mp
14. 3 T magnet.mp
15. open MRI.mp
16. MR sequence.mp
17. MR.mp
18. 8 or 9 or 10 or 11 or 12 or 13 or 14 or 15 or 16 or 17
19. 7 and 18
*EMBASE*
1. exp tooth prosthesis/
2. preformed metal crown*.mp
3. exp dental alloy/
4. stainless steel crown*.mp
5. exp mixed dentition/ or exp dentition/ or exp secondary dentition/ or exp primary dentition/
6. exp dental material/
7. 1 or 2 or 3 or 4 or 5 or 6
8. exp nuclear magnetic resonance imaging/
9. MRI.mp
10. exp functional magnetic resonance imaging/
11. functional MRI.mp
12. tesla.mp
13. 7 T magnet.mp
14. 3 T magnet.mp
15. open MRI.mp
16. MR sequence.mp
17. MR.mp
18. 8 or 9 or 10 or 11 or 12 or 13 or 14 or 15 or 16 or 17
19. 7 and 18
*Web of Science*
# 1 TS = (dentistry)
# 2 TS = (crown*)
# 3 TS = (dental alloy*)
# 4 TS = (preformed metal crown*)
# 5 TS = (stainless steel crown*)
# 6 TS = (dentition OR mixed dentition OR primary dentition OR permanent dentition OR deciduous dentition)
# 7 TS = (molar*)
# 8 TS = (dental material*)
# 9 #1 OR #2 OR #3 OR #4 OR #5 OR #6 OR #7 OR #8
# 10 TS = (magnetic resonance imaging)
# 11 TS = (MRI)
# 12 TS = (functional magnetic resonance imaging)
# 13 TS = (fMRI)
# 14 TS = (tesla)
# 15 TS = (7 T magnet)
# 16 TS = (3 T magnet)
# 17 TS = (open MRI)
# 18 TS = (MR sequence)
# 19 TS = (MR)
# 20 #10 OR #11 OR #12 OR #13 OR #14 OR #15 OR #16 OR #17 OR #18 OR #19
# 21 #9 AND #20
*Cochrane Library*
#1 MeSH descriptor: [Magnetic Resonance Imaging] explode all trees
#2 (fMRI)
#3 (MRI)
#4 (MR)
#5 (functional magnetic resonance imaging)
#6 Tesla
#7 7 T magnet
#8 3 T magnet
#9 open MRI
#10 MR sequence
#11 #1 OR #2 OR #3 OR #4 OR #5 OR #6 OR #7 OR #8 OR #9 OR #10
#12 MeSH descriptor: [Dentistry] explode all trees
#13 MeSH descriptor: [Biomedical and Dental Materials] explode all trees
#14 MeSH descriptor: [Dentition] explode all trees
#15 (preformed metal crown*)
#16 (stainless steel crown*)
#17 #12 OR #13 OR #14 OR #15 OR #16
#18 #11 AND #17

P—children aged 16 and under, undergoing MRI examination.

I—PFMC present in situ.

C—MRI examination on a patient with no PFMC.

O—degradation of image quality due to PFMC; the significant negative incident reported.

Outcome measures considered were the presence of artefact (because of PFMC) on an MR image, and the subsequent diagnostic quality of this image. Secondary outcomes were related to adverse safety incidents relating to the presence of PFMC in an MRI field.

Electronic databases were searched (MEDLINE, Embase, Web of Science, Cochrane Library) on the 9th November 2020. In addition, references of included articles were to be screened for further studies of interest. Efforts were made to identify relevant unpublished ‘grey’ literature and conference proceedings through appropriate websites and databases such as OpenGrey. Searches covered the period from the commencement of each database system until the initiation of the systematic review.

### Eligibility criteria

For studies to be included in this review, they had to meet the following inclusion criteria:

- Any primary in vivo clinical study (case series (> 10 participants), case–control, cohort, randomised and non-randomised clinical trials).
- Children under the age of 16 who had at least one PFMC placed on primary or permanent teeth that had required or undergone an MRI investigation.

For clarification, studies that included participants over the age of 16, letters and in vitro studies as well as those not written in English were excluded from this review.

### Study selection

Eligible studies were uploaded into Zotero (Version 5.0.95.1). Duplicate articles were removed. Title and abstract screening, against the inclusion and exclusion criteria, was carried out independently by two reviewers (OS & RG), with any disagreement resolved by consensus. If necessary, any unresolved differences were resolved by a third reviewer (GT).

Full texts were obtained for all titles that met these criteria. Two reviewers (OS & RG) assessed the full texts against the inclusion/exclusion criteria independently, with any disagreement resolved by consensus. If necessary, any unresolved differences were resolved by a third reviewer (GT). Reasons for exclusions were noted.

Two reviewers (OS & RG) undertook data extraction for each included study. A calibration exercise was to be conducted with all reviewers before the commencement of data extraction. Any disagreement was resolved by consensus, and where needed, any unresolved differences were resolved by a third reviewer (GT).

### Data extraction

A predesigned data extraction form will be used to extract the following data:

- Publication details: title, year, author, journal, the country in which study conducted, study design
- Aim of study
- Study characteristics: medical diagnosis; the rationale for MRI; the number of MRI scans; MRI scan characteristics, including machine and study details, area studied; the presence of artefact (as a result of PFMC) on imaging; diagnostic quality of MRI; number and age of participants; the total number of teeth with PFMC placed; type of PFMC used (including manufacturer details); the method used for placing PFMC.

### Risk of bias assessment

Risk of bias assessment was completed, independently by two reviewers (OS & RG), for each study using the ROBINS-I tool in non-randomised studies and RoB 2 tool for randomised trials (Higgins and Thomas 2020).

Like data extraction, a calibration exercise was to be conducted with all reviewers before commencement risk of bias assessment. Any disagreement was resolved by consensus, and where needed, any unresolved differences were resolved by a third reviewer (GT).

### Data synthesis

A narrative synthesis was planned to be used to explore the findings from the included studies due to the expected heterogeneity between studies.

## Results

**Fig. 1:**
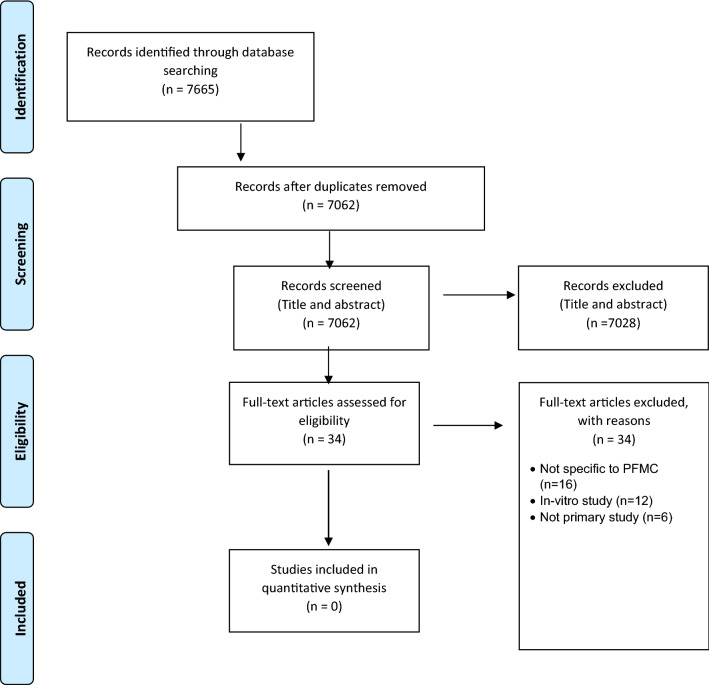
PRISMA flow chart

A total of 7665 articles were identified through searching of electronic databases. After removing duplicates, 7062 were identified for title and abstract screening. 34 articles underwent full-text review, of which none met the inclusion criteria. A summary of article selection is presented as a flowchart in Fig. 1, based on PRISMA guidelines (Moher et al. 2009).

Reasons for exclusion were varied. Sixteen studies were excluded because they did not include the placement PFMCs after closer inspection. Instead, they reported the influence cast restorations, made from various stainless-steel alloys only, had on the diagnostic quality of the MR image. Twelve studies were excluded because they were in vitro studies, and the remaining six were either narrative reviews or discussion papers.

## Discussion

To our knowledge, this is the first systematic review to attempt to evaluate the impact that PFMCs have on MRI quality and overall diagnostic value. This review followed a robust methodology and employed an extensive search strategy. The inclusion criteria were extremely broad as it was expected there would be a paucity of studies. Despite this broad approach, no studies were included for a narrative synthesis. Instead, a narrative review of the literature, including the findings from key in vitro studies, has been reported. In addition, a discussion surrounding the clinical implications and areas for future research have been provided.

PFMCs are sometimes referred to as stainless-steel crowns (SSC), the terminology is often interchangeable. PFMCs are made from 18/8 (Type 304 in the USA) stainless steel and include chromium (18–20% by weight) and nickel (8–12% by weight). The addition of chromium and nickel creates austenitic stainless steel which is non-magnetic. However, extensive cold working will alter the crystal structure of this alloy and transform it into a magnetic form (McCabe and Walls 2008). In clinical terms, there is no evidence to suggest how much adjustment (such as crimping, etc.) would be required to induce this change in the crystal structure.

An MRI investigation is non-invasive and unlike other forms of imaging, does not use ionising radiation. Instead, it uses radiofrequency, utilising the body’s natural magnetic properties to form detailed three-dimensional images. The nucleus of a hydrogen atom, abundant in water and fat, contains a single proton. These protons spin randomly around their axis in normal circumstances. In a strong magnetic field, such as is found in an MRI scanner, the proton axes all align along the magnetic field axis. Additional energy in the form of a radio wave is applied through the tissues, which deflects the protons. Once switched off, the proton returns to its original position, releasing energy in the form of another radio wave. This is then detected by receiver coils contained within the machine. Different tissue types relax at different rates, and so these differences in intensity and speed allow the various tissues to be identified and an image is built up. The field strength can be altered, causing different ‘slices’ of the body to resonate and in this way, a comprehensive three-dimensional image is formed (Berger 2002).

There are two potential concerns regarding PFMCs in an MRI field:

1. Distortion/artefact of image and impact on diagnostic quality.
2. Displacement due to the influence of the magnetic field, and the potential danger of dislodgement.

Previous studies have shown that stainless-steel alloys used in the production of cast metal prostheses cause significant distortion to MR image quality (Lissac et al. 1992; Mathew et al. 2013). Similarly, stainless-steel orthodontic appliances (e.g. brackets, bands, wire) are non-compatible with MRI, particularly where the area to be imaged is close to the appliance, as they cause significant artefact (Tymofiyeva et al. 2013; Chockattu et al. 2018; Hasanin et al. 2019). Stainless steel brackets, in particular, can cause artefact in and around oral cavity MR images, but an extension to the cerebral fossa (Tymofiyeva et al. 2013) and frontal lobes (Elison et al. 2008) have been reported. It appears that signal distortion is greatest when the area of interest of a study is within 10 cm of this metallic object (Mathew et al. 2013).

Despite causing artefact, previous reviews have concluded that well bonded fixed stainless-steel orthodontic appliances are safe for use in MRI scanners providing that the appliance is checked before the scan to ensure no attachments are loose (Chockattu et al. 2018).

Hypothetically, a PFMC could be considered an extension of an orthodontic band, and is similar in terms of size, tooth coverage, and location in the oral cavity. Anecdotally, it could be hypothesised that the conclusions, in terms of artefact and displacement, derived from the orthodontic literature could be applied to those children needing an MRI who have had PFMCs placed. However, significant differences do exist. As previously mentioned, PFMCs are made from the same stainless-steel alloy, but have less of it due to the addition of nickel and chromium. Therefore, the alloy structure is different which is likely to influence the amount of artefact produced. Additionally, metallic orthodontic appliances are placed on almost every tooth in the arch, whereas a PFMC is often confined to a single molar or even one per quadrant. It is, therefore, conceivable that this greater volume of stainless-steel products may cause a larger artefact, however, this relationship is yet to be explored in a dental context.

### In vitro studies

Several in vitro studies were identified during this review process; most, however, used different alloys to those used to make PFMCs, and so do not allow a meaningful comparison. An example of that is the study conducted by Fache et al (1987) who investigated the effects of a ‘preformed crown’ on MR image quality. Despite being the correct treatment modality, the stainless-steel alloy they used for this ‘preformed crown’ included a significantly different composition of metals, in particular chromium (15.46%) and nickel (70.42%), compared to PFMCs routinely available for use in clinical practice today (Fache et al. 1987). Interestingly, the authors reported a strong positive correlation between the magnetic permeability of a material and the degree of image distortion, with effects being limited to those materials which had a high magnetic permeability. Materials with high nickel content (> 10%) showed low magnetic permeability, but those with < 10% nickel content had even less magnetic permeability. Therefore, it could be surmised that modern day PFMCs, which have a much-reduced nickel content, should have a lower magnetic permeability, and are less likely to cause MR image distortion. However, of interest, they concluded that predicting the effects a particular dental material had on MR in each individual patient was not possible, as magnetic permeability was a function of the materials mechanical history (Fache et al. 1987). Therefore, it could be proposed that PFMCs that have required significant amount of alteration, prior to fit, or those that have undergoing distortion post-placement due to attritional wear or trauma, are more likely to cause a greater amount of image distortion.

Two in vitro studies assessed the MR image quality, with one using plates made from the same stainless-steel alloy used to make PFMCs (Lissac et al. 1991) and the other using an actual PFMC (Bryll et al. 2007). These studies both demonstrated a large image artefact made up of a central oval/circular shaped area of complete signal loss, surrounded by a zone with varying signal loss and a ‘halo’ of hyperintense signal (Lissac et al. 1991; Bryll et al. 2007).

Lissac et al (1991) went on to conduct some in vivo work by asking young adult volunteers to wear a plastic (Celluloid) prosthesis containing an 18/8 stainless-steel plate in the maxillary molar region. This produced image artefact in the maxillofacial region and extending beyond the cranium. Specifically, significant artefacts were observed in the tongue, floor of mouth and dental arches, with image deterioration of the masticatory muscles, floor of the orbit, paranasal sinuses, and nasopharynx evident. The artefact was also observed on the contralateral side (Lissac et al. 1991). These studies, and in particular the in vivo competent reported by Lissac et al (1991), provide promising estimates of the potential effect of PFMCs on MR image quality. Despite this, consideration must be made to the extent and/or spread of artefact observed in these young adults which may be different to what would be observed in a child, where a PFMC could be physically closer to the scan ‘area of interest’.

The other concern of having a metallic object in an MRI machine is the safety risk of this being displaced due to the magnetic field. Only one in vitro study investigated displacement of crowns in the magnetic field and found no such effect existed (Bryll et al. 2007). The authors conclude though that this should not be interpreted as confirmation of PFMCs safety for use in an MRI (Bryll et al. 2007).

MRI signal distortion due to a PFMC has been shown to be greatest when within 10 cm of the area of interest (Mathew et al. 2013). Anecdotally, it could, therefore, be assumed that there will be little or no impact on image quality from a PFMC in the oral cavity to MRI images conducted on other regions of the body. Furthermore, it can be assumed that MRI scans external to the head and neck region would pose even less of a risk of displacing a crown from the mouth, as the magnetic field is distant to the oral cavity, although there are no empirical studies to support this hypothesis.

### Clinical implications

Despite lacking good quality in vivo evidence, the hypothetical risk of image distortion from PFMCs on MR images is real. Detection of brain pathologies, most notably malignancies, are at risk of being harder to detect if PFMCs are in-situ. Unclear imaging, potentially because of PMFCs, could have significant implications for these children in terms of diagnosing and planning treatment, often as part of a multidisciplinary management approach. Similar issues arise for children who require regular MRIs, such as those with seizure disorders or those undergoing surveillance for benign brain malignancies. However, based on the distinct lack of clinically robust evidence, removal of PMFCs cannot be routinely indicated for every patient undergoing isolated or regular MR imaging of the head and neck region based on potential for MR image artefact. However, there will be instances where removal is indicated, and such decisions must be pragmatically made by all health care professionals involved in that child’s care.

In contrast, some patients may require a decision on how to manage their dental condition before MR imaging. This further presents a real dilemma to the paediatric dentist, as traditional restorative alternatives to PFMC’s including composite or glass ionomer restorations might be considered ‘MRI safe’ (Tymofiyeva et al. 2013) but are known to have higher failure rates (Innes et al. 2015) potentially jeopardising dental outcomes. As an alternative, a novel non-restorative, or ‘biological’ approach to dental caries management using silver diamine fluoride (SDF) could be provided. SDF is effective at arresting caries in the primary dentition (Seifo et al. 2019) with no reported concerns regarding its use in patients undergoing MRI. However, SDF (and biological techniques in general) is not appropriate for a child about to undergo oncological management, where definitive restoration or even extraction of teeth of questionable prognosis, is required (Kumar 2019). In these scenarios, if PFMCs are not able to be placed, due to concerns surrounding MRI artefact, then affected teeth may be extracted as the only ‘reasonable’ alternative—a true dilemma for any dentist when conventional wisdom extols that extraction is a ‘last resort’. It is important to recognise that these decisions should not be made in isolation. A pragmatic decision must be made that includes input from all healthcare professionals, involved in the child’s care, as well as the patient (if appropriate) and their parents.

As previously stated, well bonded fixed stainless-steel orthodontic appliances are safe for use in MRI scanners providing that the appliance is checked before the scan to ensure no attachments are loose (Chockattu et al. 2018). Despite Bryll et al (2007) reporting that PFMCs did not displace when placed in a magnetic field, it would be prudent that healthcare professionals check for any loose PFMCs prior to an MRI scan.

### Strengths and limitations

Despite not including any studies, a major strength of this review is the extensive and comprehensive literature search which was employed in relevant databases as well as an exploration of the grey literature. Furthermore, this review was willing to include any primary studies with a range of study types rather than just randomised control trials. Including such variation would likely impact the quality of data, however, given the perceived anecdotal scarcity of evidence, this would have been acceptable. As previously mentioned, further well-designed cohort or case–control studies should be undertaken to ascertain the impact PMFC has on MR images.

One major limitation was the omission of articles not written in English. It is expected that including studies published in non-English languages is likely to increase the resource challenges concerning costs, time, and expertise in non-English languages; however, their inclusion could improve generalisability and reduce the risk of systematic bias. Interestingly, Morrison et al (2012) found there to be no evidence of a systematic bias when language restrictions were placed on systematic review-based meta-analyses used for conventional medicine. Of course, this review is about medicine, however, given there is no corresponding review in dentistry, it would be sensible to apply this logic until proven otherwise.

Reviewers (OS & RG) involved in the screening process, specifically study selection and data extraction, were not blinded to either author’s names and/or study origin. This may have introduced a bias; however, it should be negligible as each reviewer worked independently and consulted with a third reviewer when disagreement was noted. The approach used for these aspects of the review conforms to the Cochrane Collaboration for Systematic Reviews of Interventions, which recommends at least two reviewers should undertake these tasks, and that blinding is not essential, as it is time-consuming and does not result in benefit or protection against bias (Higgins and Thomas 2020).

### Areas for research

It is evident from this review that there is a paucity of research in this field. This highlights an important area for future research. Well-designed studies should be undertaken to help inform practice. Of course, there may be ethical reasons why prospective experimental studies in children have not been conducted, although MRI uses non-ionising radiation and would not be harmful. Well-designed cohort or case–control studies could provide a good source of information within ethical limitations.

## Conclusion

- No in vivo studies identified in this systematic review that could inform the potential impact PFMCs would have on the diagnostic quality of children who have undergone an MRI in the head and neck region.
- From a limited number of in vitro studies, PFMC’s have the potential to cause significant image artefacts but pose a low risk of untoward or adverse effects on the patient
- Further high-quality clinical trials (e.g. cohort studies, case–control studies) should be performed to establish an evidence base
- For children who undergo regular monitoring MRI scans and require restoration of carious or worn primary teeth, then a full discussion with their medical team and radiologist about the potential risk of a non-diagnostic image caused by a PFMC. Alternative restorative techniques could be considered in this population.
